# Effect of thymoquinone on the healing of left colon anastomosis: an experimental study

**DOI:** 10.1186/s40064-016-2674-7

**Published:** 2016-07-02

**Authors:** Remzi Kızıltan, Özkan Yılmaz, Sebahattin Çelik, Serkan Yıldırm, Hamit Hakan Alp, Abbas Aras, Çetin Kotan

**Affiliations:** Department of Surgery, School of Medicine, Dursun Odabaş Medical Center, University of Yuzuncu Yil, 65090 Van, Turkey; Department of Pathology, School of Veterinary Medicine, University of Yuzuncu Yil, 65090 Van, Turkey; Department of Biochemistry, School of Medicine, University of Yuzuncu Yil, 65090 Van, Turkey

**Keywords:** Thymoquinone, Rat, Colon anastomosis, Healing

## Abstract

**Aim:**

To evaluate the effect of thymoquinone on the healing of experimental left colon anastomosis in rats.

**Methods:**

Forty Wistar albino rats weighing 250–300 g were randomly divided into four groups (10 rats/group). Group 1 (control group) rats were not administered Thymoquinone (TQ) for 3 days after the operation. Group 2 was administered daily TQ for 3 days starting from the first day after the operation. Group 3 was not administered TQ for 7 days after the operation. Group 4 was administered daily TQ for 7 days starting from the first day after the operation. Thymoquinone was administered as a single dose oral gavage through a 4F feeding catheter per each day. The bursting strength of the anastomosis was measured on 3rd and 7th postoperative days (POD) and resection was performed. Subsequently, the hydroxyproline level in the resected tissue was measured and a histological evaluation was performed.

**Results:**

The bursting pressures of the anastomoses were measured to be statistically significantly greater on 7th POD in TQ administered groups compared to those without TQ administration. Tissues were stained with Masson’s trichrome dye in order to evaluate the amount of fibrous tissue reaction for histopathological examination; there was no significant difference in the amount of fibrous tissue between groups 1 and 2, while a very marked increase in the fibrous tissue was detected in groups 3 and 4. Mean tissue hydroxyproline levels of the groups 3 and 4 on 7th POD were 1.30 and 2.72 μg/g-protein, respectively. The difference between the groups was statistically significant (p = 0.001).

**Conclusions:**

TQ significantly increased the bursting pressure of the anastomosis, tissue hydroxyproline level, and fibrous tissue production.

## Background

Colorectal anastomotic leak is a serious complication increasing postoperative morbidity and mortality (Kube et al. 2010; Frye et al. 2009). It is important to prevent or decrease the rate of such leaks.

The antioxidant, anti-inflammatory and anti-cancerous effects of thymoquinone, an active form of *Nigella sativa*, have been investigated both in vitro and in vivo since the 1960s. Its antioxidative and anti-inflammatory effects have been established in some disease models such as encephalomyelitis, diabetes, asthma and carcinogenesis (Woo et al. 2012). Some studies have been published in the literature exploring the effects of various agents such as *Ginkgo biloba* extract (EGb 761), glucocorticoids, and beta-glucan on the healing of colonic anastomosis (Kisli et al. 2007; Ostenfeld et al. 2015; Caglayan et al. 2013). However, no study investigating the effects of thymoquinone was encountered in the literature. Based on the antioxidative, anti-inflammatory and immunomodulation effects of thymoquinone, the present study aimed to investigate its effects on the anastomosis of the left colon that usually has a thinner wall and a higher anastomotic risk.

## Methods

### Animals

Each groups in this study was intended to contain ten rats considering the possibility of losing some rats from surgical complications in the postoperative period. Forty, 4-month-old, female Wistar albino rats weighing 250–300 g were included in the study. All animals were treated humanely in accordance with Declaration of Helsinki. The rats were divided into four groups following the operation (n = 10).

Group 1: Control group that was not administered thymoquinone for three postoperative (PO) days
Group 2: Group that was administered thymoquinone for three PO days
Group 3: Control group that was not administered thymoquinone for seven PO days
Group 4: Group that was administered thymoquinone for seven PO days

### Operative procedures

No mechanical or antibacterial bowel preparation was applied in the rats. In all rats anesthesia was applied using intramuscular Ketamine hydrochloride (Ketalar, Eczacibasi, Istanbul, Turkey) at a dose of 75 mg/kg following 8 h of fasting. Subsequently, an abdominal midline incision was used to enter the abdominal cavity. The left colon was found and the sigmoid colon was transected at 3 cm above the peritoneal reflection and an end-to-end single layer anastomosis was performed using a 6/0 polypropylene (6–0 monofilament polypropylene; Prolene, Dogsan) suture. All anastomoses were performed by the same surgeon using the same technique. Abdominal muscle layers and skin were closed with single sutures of 3/0 silk (3/0 silk, Dogsan) sutures.

### Thymoquinone administration

Thymoquinone (code:274666 SIGMA**-**ALDRICH) 50 mg/kg/day was administered in a 1 ml single dose oral gavage dissolved in olive oil through a 4F feeding catheter each morning starting from postoperative day 1. It was administered as daily doses starting from the first postoperative day in groups 2 and 4. The rats were fed normal diet and water ad libitum. Rats in the group in which Thymoquinone was not administered were fed normal diet and water ad libitum.

### Bursting pressure

**Fig. 1:**
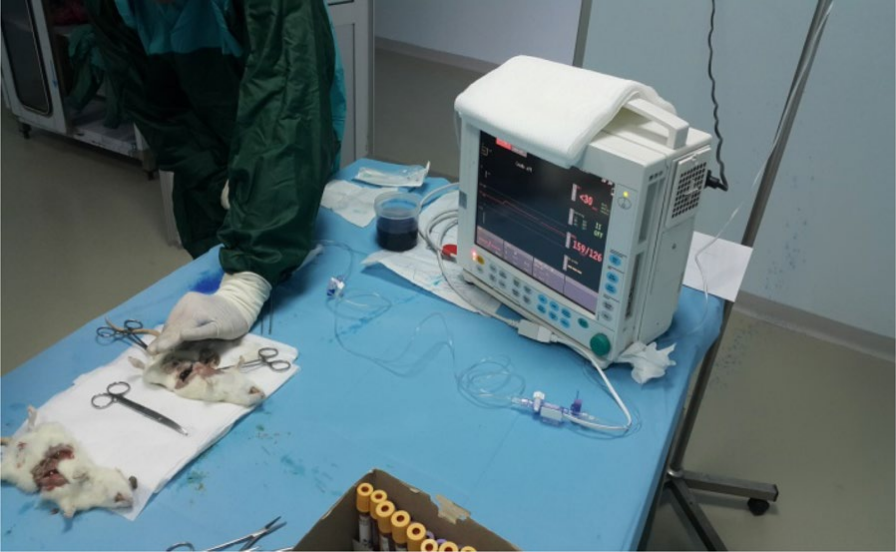
Anastomosis bursting pressure measurement

Rats were administered anesthesia on 3rd and 7th POD using the same technique. The abdominal incision was subsequently opened. The colon lumen was obstructed using a clamp at a level 5 cm proximal to the anastomosis without removing any adhesions over the anastomosis. Arterial set connected to a monitor with the trademark Datex-Ohmeda (Compact Monitor; Type: S5; Manufacturer: Datex-Ohmeda GE Healthcare) was placed in the anal canal of the rats and calibration was performed. Subsequently, another catheter to be used for the introduction of serum physiologic dyed with methylene blue to increase the intracolonic pressure was placed in the anal canal. A solution of serum physiologic dyed with methylene blue was administered intracolonically at a rate of 2 ml/min using an infusion pump. Pressure changes inside the colon were observed instantaneously. The first time when a blue dyed leak from the anastomotic line was observed, the pressure was recorded as the anastomotic bursting pressure (Fig. 1).

### Analyses

Anastomotic bursting strength was measured as stated above. Subsequently, colonic resection was performed at a level 5 cm above and 5 cm below the anastomosis. The anastomotic line was divided into two pieces using a vertical incision performed at the midline of the anastomotic line. One half was collected for the measurement of hydroxyproline, a marker of tissue collagen content (Agyare et al. 2016) and the other half was collected for histopathological examination.

### Determination of hydroxyproline level

We used the method described by Hutson et al. for hydroxyproline analysis. Sample rat intestines were stored at −80 °C and the wet weight of each sample was recorded. The tissue samples was homogenized in 1 ml of 6 N HCI with a mechanical homogenizer. Then, 200 µl of homogenate was placed in a clean glass test tube and 3.8 ml 6 N HCI was added. 100 ml of 2 mM sarcosine for the 5 in water were added to each tube, after which the tubes were 2.1. Materials were tightly capped and placed in a 110 °C heating block for 18 h. The hydrolysates were allowed to cool down to room temperature and neutralized with 4 ml of 6 M NaOH. Each sample was brought to a pH of 9.56 ± 1.0 with 6 M NaOH. Aliquots of 900 ml of this solution were removed for the subsequent derivatization process. Derivatization procedure was the same as described by Hutson et al. (2003).

### Histopathological analyses

Following the necropsy, tissues obtained for histopathological evaluation were fixated in 10 % formalin solution for 48 h and then washed under flowing tap water. They were passed through routine tissue follow-up in alcohol (70°, 80°, 90°, 96°, and 100°) and xylol series and then embedded in paraffin blocks. Sections measuring 4 μm thick were taken from each block and the preparations were made on microscope slides. Preparations for histopathological examination were dyed with hematoxylin-eosin (HE) and with Masson’s trichrome stain in order to achieve a more marked evaluation of the fibrous tissue and then were examined under the light microscope (Leica DM 1000, Germany).

### Statistical analysis

SPSS (IBM Corp. Released 2013. IBM SPSS Statistics for Windows, Version 22.0. Armonk, NY: IBM Corp.) statistical software package was used for all statistical analyses. The Wilcoxon test was used to evaluate the difference between the bursting pressures (mmHg) of the rats in the groups with and without TQ administration. The Mann–Whitney U-test was also used to evaluate the differences between the groups, and the hydroxyproline levels measured in the intestinal tissue of the rats (gr/gr-protein). Level of statistical significance was accepted as 5 % for all tests.

### Results

A total of seven rats died on first postoperative day (2 in group 1, 3 in group 2, and 1 each in groups 3 and 4). This may be due to the anesthetic reactions. The deceased rats were not included in the measurements. Anastomotic leak or wound dehiscence was not detected in any of the rats.

### Anastomotic bursting pressure findings

**Fig. 2:**
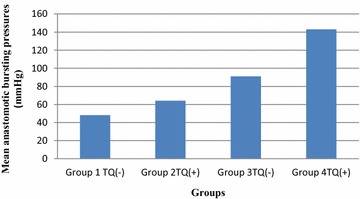
Mean anastomotic bursting pressures

Anastomotic bursting pressures were measured to be statistically significantly higher in TQ administered groups compared to those with no TQ administration on seventh postoperative day (p = 0.008). Bursting that occurred outside the anastomosis region occurred in six of nine rats due to high pressure in group 4 (TQ administered on 7th POD) (Table 1; Fig. 2).

**Table 1:** Anastomotic bursting pressures (mmHg)

	Group 1 TQ (−) mmHg	Group 2TQ (+) mmHg	Group 3TQ (−) mmHg	Group 4 TQ (+) mmHg
1	13	45	108	130
2	61	59	118	148^a^
3	68	76	111	130^a^
4	37	51	40	121^a^
5	32	82	93	146^a^
6	59	49	106	163^a^
7	66	86	96	127
8	57		86	150
9			66	176^a^
Mean	48 ± 20.8	64 ± 16.9	91 ± 24.8	143 ± 18.1
p value	0. 091		0.008	

p < 0.05 indicates statistical significance, Wilcoxon Test were used for statistical analysis

^a^Bursting occurred outside the anastomosis

### Findings of tissue hydroxyproline levels

**Fig. 3:**
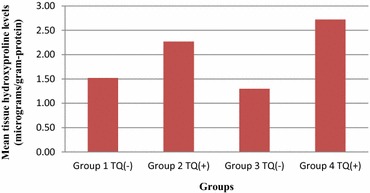
Mean tissue hydroxyproline levels

Tissue hydroxyproline levels were higher in TQ administered groups. The results are presented on Table 2. The differences between groups 1 and 2 (p = 0.010) and groups 3 and 4 (p = 0.001) were statistically significant (Table 2; Fig. 3).

**Table 2:** Tissue hydroxyproline levels (micrograms/gram-protein)

	Group 1 TQ (−)	Group 2 TQ (+)	Group 3 TQ (−)	Group 4 TQ (+)
1	1.10	2.38	1.11	1.81
2	1.28	1.79	1.61	2.35
3	1.62	2.93	1.28	2.89
4	1.58	2.17	1.25	2.94
5	1.46	1.82	1.16	3.25
6	1.41	2.56	1.10	3.30
7	1.84		1.25	2.71
8	1.90		1.16	2.53
9			1.86	
Mean	1.52 ± 0.26	2.27 ± 0.44	1.30 ± 0.25	2.72 ± 0.49
p value	0.010		0. 001	

p < 0.05 indicates statistical significance, Mann–Whitney U Test were used for statistical analysis

## Histopathological results

### Macroscopic findings

The macroscopic evaluation revealed an edematous, swollen, and hyperemic anastomotic line in the first group. Also, a fibrous inflammation was present, extending 1 cm right and left of the anastomotic line. In the second group, there was a grayish white line on the anastomotic line, but the fibrous tissue was only present around the wound.

The seventh day evaluation revealed a grayish white fibrous tissue on the anastomotic line in the third group and the colon lumen was found to be narrowed. A macroscopic evaluation of the fourth group revealed that this fibrous tissue was shiny white and quite thin.

### Microscopic findings

Upon microscopic evaluation, an intense mononuclear cell infiltration on the anastomotic line was observed in the first group. Hyperemic and hemorrhagic foci were present in the vascular structures and necrotic and degenerative changes were present in the colonic mucosa and there was an exudate including bacterial clusters.

An evaluation of the second group revealed lesions in the anastomotic line, similar to the first group and a marked revascularization, while fibrosis was found not to have started yet.

The seventh day evaluation demonstrated fibrous tissue proliferation and mononuclear cell infiltration here and there in the third group. Regeneration was detected in the epithelial layer. A mature fibrous tissue was seen in the examination of the fourth group and the necrotic mass was seen to be completely removed.

**Fig. 4:**
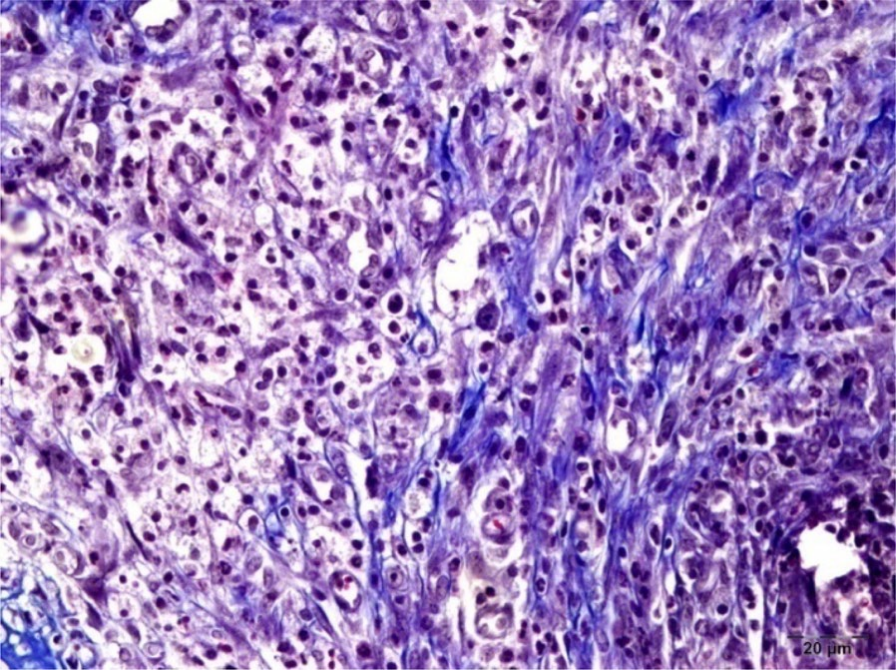
Connective tissue proliferation in the anastomotic line in group 3, Masson’s trichrome, *bar* 20 µ

**Fig. 5:**
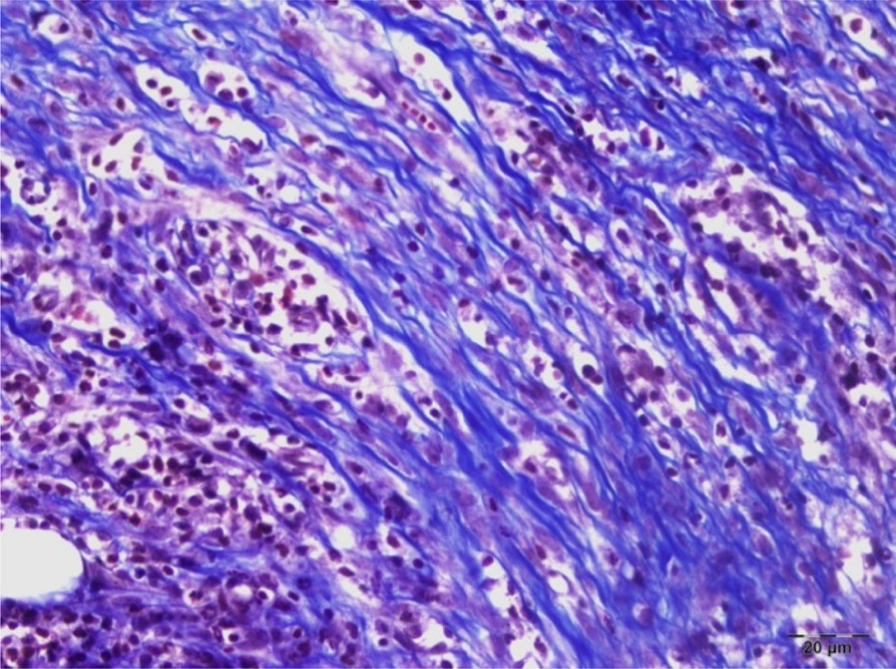
Severe connective tissue proliferation in the anastomotic line in group 3, Masson’s trichrome, *bar* 20 µ

The tissues were stained with Masson’s trichrome dye in order to evaluate the fibrous tissue reaction and they were scored as described on Table 3. Scoring was performed as follows: no fibrosis formation: 0; very little activity: 1; a marked connective tissue proliferation: 2; and severe and mature connective tissue proliferation: 3 (Table 3; Figs. 4, 5).

**Table 3:** Fibrosis severity scoring, microscopic

Group	Score
1. Group	0–1
2. Group	0–1
3. Group	2
4. Group	3

The amount of the connective tissue was similar in groups 1 and 2, while a very marked difference in the increase in connective tissue was found between groups 3 and 4.

## Discussion

The rate of anastomotic leaks has been reported to be 3–6 and 10–12 % following colonic and rectal resections, respectively (Peeters et al. 2005; Krarup et al. 2012). This condition increases mortality and morbidity. Therefore, anastomotic leaks is a source of great concern among surgeons. Therefore, surgeons have been seeking new methods of treatment to prevent these leaks.

The inflammatory process is one of the ordinary healing phases of the intestinal wall (Enoch and Leaper 2005; Wu et al. 2015). In this process, coordination is necessary between the cellular activity and humoral factors (Inan et al. 2006). The degree of inflammatory response, the rate of mucosal re-epithelization and the amount of newly synthesized collagen directly affect the anastomotic healing. The durability of the anastomosis is associated with the amount of collagen fibrils in the submucosal layer and their maturation (Aslan et al. 2008). Fibroblast proliferation and collagen synthesis occur in the submucosal layer (Inan et al. 2006). Fibroblasts maximally synthesize collagen on the fifth to seventh days postoperatively. Therefore, the present study ended on the seventh day, as in previous studies in the literature (Aslan et al. 2008; Kosmidis et al. 2011).

Kosmidis et al. demonstrated the key role of myofibroblasts in the healing process of the anastomosis following colon surgery for cancer and reported that the rate of anastomosis breakdown decreased following the seventh day (Kosmidis et al. 2011).

Anastomotic bursting strength and perianastomotic tissue hydroxyproline level has been used in experimental studies to evaluate the integrity of anastomosis (Inan et al. 2006). Anastomotic bursting strength, tissue hydroxyproline level at the anastomotic region and histopathological evaluations of tissue samples from the anastomotic line were used in the present study to evaluate the effect of TQ on the anastomotic healing.

The integrity and quality of the anastomosis are associated with the amount of collagen synthesized (Kuper et al. 2011). On the other hand, hydroxyproline is a major component of collagen. In the present study, hydroxyproline levels in the tissue obtained from the anastomotic line were statistically significantly higher in TQ administered groups compared to the groups without TQ administration. Based on this result, it can be suggested that TQ increases collagen synthesis.

The histopathological examination of the anastomotic line did not reveal any marked increase in the connective tissue formation between 3-day groups, which were groups 1 and 2, while a very marked difference in the increase in connective tissue was found between groups 3 and 4. In the fourth group with 7th POD and TQ administration, the increase in connective tissue was found to be very marked.

Many studies have been performed to increase the anastomotic integrity and to reduce the rate of leaks. In a systematic review performed by Oinen et al., anastomotic bursting strength increased by 60 mmHg with iloprost, a prostacyclin analogue; by 48 mmHg with large spectrum metalloproteinase inhibitors; 45 mmHg with erythropoietin; 29 mmHg with tacrolimus, an immunosuppressive drug and 24 mmHg with hyperbaric oxygen therapy (Oines et al. 2014). In the present study, the mean anastomotic bursting strength was 91 mmHg in group 3 on 7th postoperative day, while the mean anastomotic bursting strength was 143 mmHg in group 4. TQ increased the anastomotic bursting strength by 52 mmHg on 7th postoperative day. This was statistically significant (p = 0.008). On the other hand, bursting site was outside the anastomotic region in six of nine rats in the 7th POD group administered TQ. Based on these findings, we can conclude that TQ is a very effective agent on anastomotic healing.

TQ is a phytochemical with powerful antioxidative properties. The study of Umar et al. demonstrated that TQ caused an increase in the activity of the antioxidative enzymes, glutathione (GSH), catalase (CAT) and superoxide dismutase (SOD). It also showed that it inhibited the increase in nitric oxide (NO) and myeloperoxidase (MPO) (Umar et al. 2012). TQ collects the reactive oxygen radicals such as superoxide anion and hydroxyl radical (Mansour et al. 2002; Nagi and Mansour 2000). Therefore, it can antagonize the effects of reactive oxygen radicals. In a study by Houghton et al., TQ was demonstrated to inhibit the production of eicosanoids such as thromboxane B2 and leukotriene (LT) B4, and thus was found to have an anti-inflammatory effect (Houghton et al. 1995). TQ inhibits both cyclooxygenase and 5-lipoxygenase enzymes (Houghton et al. 1995). Also, TQ significantly inhibits LTC4 synthase (Mansour and Tornhamre 2004). Another study demonstrated a decrease in the levels of TNF-alpha, interleukin-6 (IL-6) and IL-1 in blood and tissues and also a decrease in inflammation and protection of tissues (Tekeoglu et al. 2006; Vaillancourt et al. 2011). TQ also has an immunomodulatory effect. It was demonstrated to inhibit IL-6 production and to decrease the secretion of cytokines such as IL-1 beta and IL-8 in mixed lymphocyte cultures (Salem 2005; Gholamnezhad et al. 2015). Based on these effects, it can be suggested that TQ decreases mucosal and submucosal damage and edema.

The inflammatory mediators and immunomodulation are reported to be essential in tissue healing process (Salem 2005). As our study demonstrated, it can be assumed that the anti-inflammatory and immunomodulator effect of TQ can be a result of its action on the anastomotic healing. In a study by Bozdag et al., intraperitoneally applied TQ was shown to decrease the hydroxyproline level and intraabdominal adhesions (Bozdag et al. 2015). In the present study, orally administered TQ was demonstrated to increase the hydroxyproline levels on the anastomotic line. A better integrity and quality of the anastomosis brought by the effect of TQ was shown by bursting strength and histopathological findings.

## Conclusion

Anastomotic leak is one of the long standing major problems for surgeons. TQ significantly increased the bursting strength of the anastomosis, tissue hydroxyproline levels and connective tissue production. As a result of the present study which is first in the literature on anastomotic healing performed using TQ, it can be concluded that TQ decreases mucosal and submucosal damage and edema and increase collagen synthesis.
